# Intelligence

**Published:** 1809-04

**Authors:** 


### INTELLIGENCE.

On Wednesday the 8th of March, the MEDICAL SOCIETY of LONDON, held their Anniversary Meeting at their house in Bolt Court, Fleet Street, when the following Officers and Council were elected for the year ensuing.

PRESIDENT, Dr. Lettsom.

VICE PRESIDENTS, Dr. Bancroft, Dr. Babington, Mr. Norris, and Mr. Ware.

TREASURER, Dr. Sayer Walker.

LIBRARIAN, Dr. Clutterbuck.

SECRETARIES, (Foreign Correspondence) Mr. J. M. Good, Dr. Hamilton, Dr. Poignand.

REGISTRAR, Mr. A. B. Turnbull.

MEMBERS OF THE COUNCIL, Mr. Field, Mr. Chamberlaine, Mr. Hooper, Mr. Hurlock, Dr. Haighton, Mr. Abernethy, Sir John Hayes, Bart. Dr. Adams, Mr. Ring, Dr. Bradley, Mr. Seaton, Dr. Thornton, Mr. Griffith, Mr. Heaums, Mr. Robinson, Mr. Astley Cooper, Dr. R. Pearson, Mr. Headington, Mr. Taunton, Mr. M'Donald, Mr. Hopkins, Mr. Young, Mr. Platt, Mr. Addington, Dr. Lidderdale, Mr. Ramsden, Dr. E. N. Bancroft, Mr. Andree, Mr. Lawrence, Mr. Shearman.

To deliver the Anniversary Oration for 1810, Dr. Birkbeck.

After the Election, Dr. Sayer Walker delivered the Anniversary Oration, on the best means of promoting Medical Science. A numerous body of the Fellows of the Society afterwards dined together at the London Coffee House.

The Council of the Society have lately come to the resolution of publishing, at short intervals, and with as much regularity as circumstances will admit, a Selection from the Communications laid before the Society, instead of waiting, as heretofore, for materials to complete a volume. By this plan they hope to produce a more ready communication of practical improvements discovered by individuals.

---

To some enquiries respecting the smallest number of Galvanic combinations, and the smallest surface of plates that is sufficient to decompose the fixed alkalis; and also, the best solution for charging a battery so as to produce the greatest power, Professor Davy has given the following answers.—"In my early experiments upon potassium, I often procured it by means of a battery of one thousand pairs of plates of copper and zinc of six inches square, charged with a solution of concentrated nitrous acid in about forty parts of water. This is the lowest power that I employed; but as some of the plates had been much corroded by former processes, I should conceive that a combination of eighty would would be sufficient, provided the whole arrangement was perfect. The decomposition of alkaline earths and ammonia by amalgamation or combination of their bases may be accomplished by a much weaker combination, fifty plates of six or four inches square being adequate to produce sensible results. The potassium which I have used in various analytical enquiries lately carried on, has been all procured by chemical means, without the application of electricity. Potash may be decomposed by different processes, some of which are described in a paper which I am now reading before the Royal Society; but the best method is that which we owe to the ingenious researches of Messrs. Gay, Lussac, and Thenard, and which is the first of this kind, by mere chemical attraction, made known. When melted potash is slowly brought into contact with iron turnings or filings, heated to whiteness, hydrogen gas is evolved, holding potassium in solution: and if one part of the iron tube or gun-barrel in which the experiment is made, be preserved cool, the metal is deposited in this part, being precipitated from the hydrogen gas by cooling. The potassium is never procured quite so pure in this way as by electricity; but it is fit for analytical purposes, and I have obtained it with so little alloy, as to possess a specific gravity considerably below 8, water being 10. I have now by me a compact mass produced in an operation, which weighs nearly 100 grains."

Dr. Ramsbotham will commence a Summer Course of Lectures on the Science and Practice of Midwifery, and on the Diseases of Women and Infants, on Monday, May 8, at ten o'clock in the morning, at his house, No. 9, Old Jewry. The descriptive and physiological parts of these Lectures are elucidated by a reference to appropriate specimens of anatomical preparations from a very celebrated Collection; and the practical parts are taught upon a well constructed machine, and by attendance upon cases, when the pupil is properly qualified.

Dr. Squire will, on Tuesday, April 11, begin a Course of Lectures on the Theory and Practice of Midwifery, and the Diseases of Women and Children.

Mr. Martin, who has been diligently employed in the study of extraneous fossils for some years back, is about to publish, under the patronage of Sir Joseph Banks, a 4to Volume of Plates and Descriptions of the Petrefactions of Derbyshire. A work, by the same author, has just been printed off, containing an Elementary Introduction to the Knowledge of Extraneous Fossils; an attempt to establish the study of these bodies on scientific principles. It forms an 8vo volume, and will be given to the public in the course of the month.

A System of Surgery, will soon appear in four volumes, 8vo. by Mr. Russell, of Edinburgh.

There is also in the press another System of Surgery, of the same size, by Dr. John Thomson, Professor of Surgery to the

Royal

Royal College of Surgeons, and Regius Professor of Military Surgery, in the University of Edinburgh.

